# *sparsesurv*: a Python package for fitting sparse survival models via knowledge distillation

**DOI:** 10.1093/bioinformatics/btae521

**Published:** 2024-08-23

**Authors:** David Wissel, Nikita Janakarajan, Julius Schulte, Daniel Rowson, Xintian Yuan, Valentina Boeva

**Affiliations:** Department of Computer Science, ETH Zurich, Zurich, 8092, Switzerland; Department of Molecular Life Sciences, University of Zurich, Zurich, 8057, Switzerland; SIB Swiss Institute of Bioinformatics, Lausanne, 1015, Switzerland; Department of Computer Science, ETH Zurich, Zurich, 8092, Switzerland; IBM Research Europe, Zurich, 8803, Switzerland; Department of Computer Science, ETH Zurich, Zurich, 8092, Switzerland; Department of Computer Science, ETH Zurich, Zurich, 8092, Switzerland; SIB Swiss Institute of Bioinformatics, Lausanne, 1015, Switzerland; Department of Computer Science, ETH Zurich, Zurich, 8092, Switzerland; Department of Computer Science, ETH Zurich, Zurich, 8092, Switzerland; SIB Swiss Institute of Bioinformatics, Lausanne, 1015, Switzerland; Université de Paris, UMR-S1016, Institut Cochin, Paris, 75014, France

## Abstract

**Motivation:**

Sparse survival models are statistical models that select a subset of predictor variables while modeling the time until an event occurs, which can subsequently help interpretability and transportability. The subset of important features is often obtained with regularized models, such as the Cox Proportional Hazards model with Lasso regularization, which limit the number of non-zero coefficients. However, such models can be sensitive to the choice of regularization hyperparameter.

**Results:**

In this work, we develop a software package and demonstrate how knowledge distillation, a powerful technique in machine learning that aims to transfer knowledge from a complex teacher model to a simpler student model, can be leveraged to learn sparse survival models while mitigating this challenge. For this purpose, we present *sparsesurv*, a Python package that contains a set of teacher–student model pairs, including the semi-parametric accelerated failure time and the extended hazards models as teachers, which currently do not have Python implementations. It also contains in-house survival function estimators, removing the need for external packages. *Sparsesurv* is validated against R-based Elastic Net regularized linear Cox proportional hazards models as implemented in the commonly used glmnet package. Our results reveal that knowledge distillation-based approaches achieve competitive discriminative performance relative to glmnet across the regularization path while making the choice of the regularization hyperparameter significantly easier. All of these features, combined with a sklearn-like API, make *sparsesurv* an easy-to-use Python package that enables survival analysis for high-dimensional datasets through fitting sparse survival models via knowledge distillation.

**Availability and implementation:**

*sparsesurv* is freely available under a BSD 3 license on GitHub (https://github.com/BoevaLab/sparsesurv) and The Python Package Index (PyPi) (https://pypi.org/project/sparsesurv/).

## 1 Introduction

Survival analysis, or modeling the time until an event of interest occurs, has been one of the primary statistical tools utilized by clinicians and researchers to determine potential associations of covariates with patient survival (Kalbfleisch and Prentice 2011). One of the most widely used models for survival analysis is the Cox Proportional Hazards (Cox PH) due to its comprehensible mechanism of linking covariate effects to the hazard and survival functions (Breslow 1972, Cox 1972). Publicly available cancer cohorts, such as The Cancer Genome Atlas (TCGA), offer molecular data beyond standard clinical characteristics, such as transcriptomic and epigenomic data, to be analyzed using survival analysis methods (Weinstein *et al.* 2013). However, due to the large dimensionality of these molecular data, cancer datasets usually have more covariates than patient samples; thus, their analysis requires proper regularization to prevent overfitting. To circumvent this issue and investigate the connections between patient survival and covariates, regularized approaches, such as the Lasso, have been developed for survival analysis, with the additional aim of achieving improved interpretability and ease of use (Tibshirani 1996, 1997).

Linear regularized methods have contributed to significant advances in survival analysis. However, they are not without their shortcomings. One notable issue is their sensitivity to the choice of regularization hyperparameters, potentially leading to suboptimal performance compared to non-sparse regularized methods like Ridge regression (Ching *et al.* 2018). Consequently, it is crucial to develop interpretable and user-friendly approaches that can reproduce the benefits of existing Elastic Net implementations and regularized Cox PH models like glmnet while addressing some of their limitations. Existing R packages for sparse survival models tend to focus on specific model classes, such as the Cox PH model in glmnet (Friedman *et al.* 2010, Simon *et al.* 2011) and grpreg (Breheny and Huang 2009, Breheny 2015, Breheny and Huang 2015), or the Accelerated Failure Time (AFT) model in penAFT (Suder and Molstad 2022). This creates a need for a unifying approach allowing users to train multiple models within one package.

Our implementation is directly inspired by the pre-conditioning approach described by Paul *et al.* (2008), which would likely be termed Knowledge Distillation (KD) today. In their work, Paul *et al.* (2008) pointed out the good performance of their pre-conditioning approach when applied to Cox PH models, but it was not rigorously benchmarked against sparse Cox PH models that are fit with regularizers like the Lasso. Throughout this work, we refer to our implemented methods as using KD since we believe this term is more familiar to most readers in the bioinformatics and machine learning communities. We emphasize, however, that the original proposal for fitting sparse semi-parametric survival models in this manner goes back to the proposal of pre-conditioning by Paul *et al.* (2008).

Here, we propose *sparsesurv*, an easy-to-use Python package that enables survival analysis for high-dimensional datasets while making the choice of the regularization hyperparameter significantly easier. Our package has two main components. First, we implement variants of the AFT and Extended Hazards (EH) models, each based on kernel-smoothing the profile likelihood (Zeng and Lin 2007, Tseng and Shu 2011). Second, we present an easy-to-use pipeline that enables fitting sparse survival models via KD. We implement efficient and numerically stable scoring functions in the form of corresponding model likelihoods and survival function estimators for the Cox PH with Breslow and Efron tie corrections, AFT, and EH models. Through experimental validation, we demonstrate the utility of KD as a valuable tool for sparse survival models. We focus on the Cox PH model as an illustrative example, showcasing how KD can, in some cases, enhance discriminative performance across sparsity levels and simplify the process of selecting the optimal regularization hyperparameter.

## 2 Materials and methods

In our experiments, we focus exclusively on right-censored survival analysis (i.e. we disregard truncation and other censoring schemes). The terms “right-censored survival analysis” and “survival analysis” will be used interchangeably hereafter.

### 2.1 Knowledge distillation in *sparsesurv*

The KD framework separates the process of model estimation from feature selection. It first estimates a model that approximates the outcome well and then estimates a sparse approximation of this first model (Hinton *et al.* 2015, Stanton *et al.* 2021). Our implementation of KD thus consists of two steps. First, a teacher model is fitted to the time-to-event training dataset using the assumptions of an appropriate survival model. The teacher then makes predictions on the entire training dataset. In the second step, these teacher-generated predictions serve as targets to fit the student with a linear regression that is regularized with an appropriate penalty to encourage coefficient sparsity. Our package provides a general class, KDSurvCV, that enables the effective separation of teacher and student via sklearn pipelines (Pedregosa *et al.* 2011, Buitinck *et al.* 2013).

### 2.2 Implementation details

All functions of *sparsesurv* are implemented in Python. We use just-in-time compilation via numba for all performance-critical aspects, particularly the calculation of gradients, loss functions, and appropriate survival function estimators (Lam *et al.* 2015). We leverage the Application Programming Interface (API) of celer as the sparse regression solver in *sparsesurv* (Massias *et al.* 2018). *Sparsesurv* uses type hints and is tested using pytest. We implemented our package following the scikit-learn API, which enables the usage of all models with pipelines and other scikit-learn-related features, such as calling fit on the model object to train and predict for inference. Our package is currently installable from PyPi and GitHub. We refer to the Supplementary material for further details on the implementation of *sparsesurv*.

### 2.3 Experiments

To validate our KD version of the Cox PH model, we performed experiments on 10 transcriptomic datasets from TCGA and compared it to the Elastic Net regularized Cox PH implementation in glmnet. In particular, we used the TCGA datasets from prior work on predicting survival from RNA-seq (Ching *et al.* 2018). We focus on the Cox PH model since it is arguably the most commonly used survival model and likewise, glmnet is a commonly used R package to fit survival models. We benchmark both a linear Breslow teacher (Breslow KD) and a neural network-based Breslow teacher (KD Cox-Nnet) for *sparsesurv*. glmnet, on the other hand, only offers the Breslow approximation and so is only benchmarked on this method for performance comparison. We also included a version of glmnet that tunes the L1 ratio (glmnet tuned (Breslow)) and a version of the *sparsesurv* Breslow KD model that chooses the regularization hyperparameter to favor sparser models (Breslow KD (pcvl)). We refer to Supplementary Methods and Supplementary Table S1 for further clarity on datasets and Supplementary Methods for experimental details.

## 3 Results

**Figure 1.**
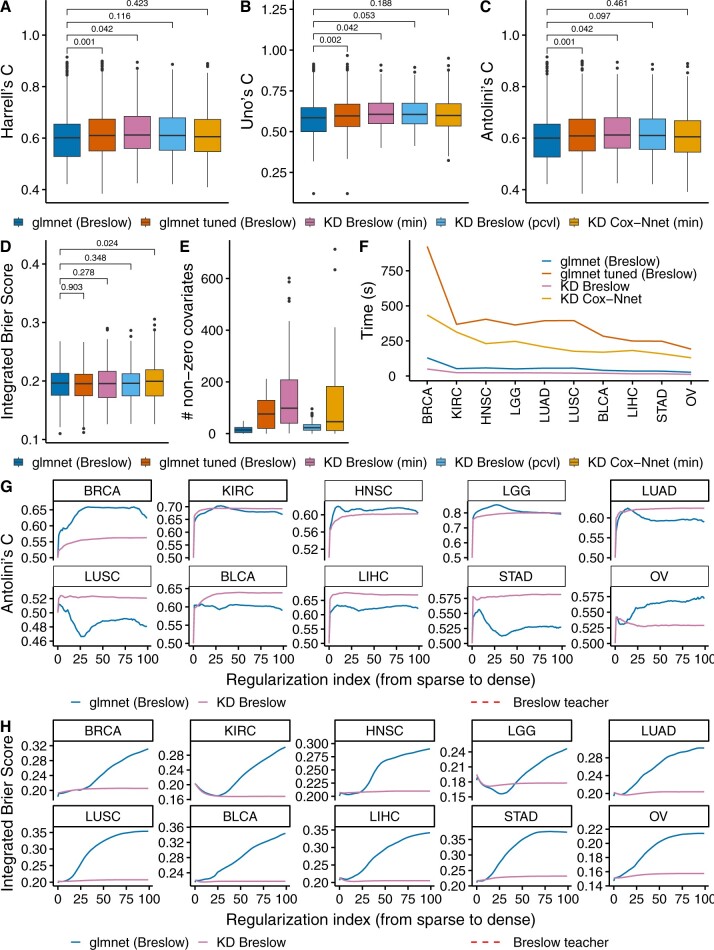
The distilled Elastic Net-regularized Cox PH Breslow model (KD Breslow (min)) performed competitively with a non-distilled Breslow model (glmnet Breslow) in a comparison between *sparsesurv* and glmnet for sparse Cox PH models on 10 TCGA datasets. The teacher model corresponds to a linear maximum likelihood model with the Breslow tie correction that was trained on a PCA representation obtained from the original datasets (Breslow teacher; Supplementary Methods). Each boxplot represents performances across a 5-fold CV on each dataset of 10 datasets, five times repeated, yielding a total of 250 points in each boxplot. (A–C) The distilled models performed comparably with the non-distilled Cox PH model trained using glmnet in terms of most concordance measures across all 10 datasets. (D) The KD models exhibited similar model calibration to the non-distilled Cox PH models. (E) The non-distilled model exhibited greater sparsity when selecting the regularization hyperparameter that minimizes overall cross-validation error. While the tuned glmnet model also slightly outperformed the untuned glmnet model, it required more variables than a comparable KD model (glmnet tuned (Breslow) vs KD Breslow (pcvl)). (F) The distilled models were considerably faster than non-distilled models since they can rely on highly optimized sparse solvers for linear regression, such as celer. We do not show both the min and pcvl choices to choose regularization hyperparameters, since they have practically no impact on runtime. (G,H) The distilled model was more stable in terms of performance than the non-distilled model broadly across the regularization path for most cancer types. This simplifies the selection of the regularization hyperparameter, allowing for more flexibility in inducing specific sparsity levels. “Regularization index” denotes the ordering of the lambda on the regularization path, where zero indicates the first that encourages complete sparsity and 99 indicates the last that encourages minimum sparsity, that is, in our case (see Supplementary Methods). All *P*-values were calculated with one-sided paired Wilcoxon signed-rank tests. For concordance measures, we applied one-sided statistical tests for the superiority of each model compared to glmnet (Breslow), while for the Integrated Brier Score, we applied one-sided statistical tests for the superiority of glmnet (Breslow) compared to each other model. See Supplementary Methods for further details on statistical significance testing. Confidence bands denote one standard error deviation to either side where applicable. TCGA, The Cancer Genome Atlas; KD, knowledge distillation. For cancer-type abbreviations, refer to Supplementary Table S1.

The distilled methods implemented in *sparsesurv* show competitive performance relative to non-distilled models (Fig. 1). In particular, the distilled models (KD Breslow (min) and KD Breslow (pcvl)) slightly outperformed, on average, non-distilled models (glmnet (Breslow)) in terms of Harrell’s, Uno’s concordance, and Antolini’s concordance (Fig. 1A–C), although the improvement was not always statistically significant. When tuning the L1 ratio of the glmnet model, it also performed better than the untuned model, although this came at the cost of decreased sparsity (Fig. 1A–C, E). For the Cox-Nnet teacher, the calibration score showed a small decrease as a result of distillation as measured by the IBS. Otherwise, distilled models and undistilled models performed comparably (Fig. 1D). We observed that distilled Cox PH models were considerably less sparse (Fig. 1E and Supplementary Table S2) compared to the glmnet model, which encountered numerical issues several times and was prone to selecting completely sparse models (Supplementary Table S3). Despite this, KD Breslow (pcvl) was still more sparse than the tuned glmnet model, while achieving comparable prediction performance (Fig. 1A–D). We also found that the distilled models in *sparsesurv* were considerably faster than glmnet (Fig. 1F and Supplementary Table S4). The high speed of distilled models can be primarily attributed to the efficient implementations in celer for sparse student models (Massias *et al.* 2018).

The teachers overall performed similarly to their students, with both types of model slightly outperforming the untuned for the L1-ratio glmnet model in terms of the three concordance measures but not the Integrated Brier Score (IBS) (Supplementary Fig. S1). The Cox-Nnet student behaved similarly to the student of the PCA-based linear Cox model, with its performance converging to the teacher performance as regularization was lessened, although convergence for the IBS was much more variable relative to the student of the linear teacher (Fig. 1G and H and Fig. S2). Lastly, we investigated the impact of performing a greater number of cross-validation repetitions (25 instead of five, see Supplementary Methods) and calculating the concordance measures across all test splits of a CV jointly instead of individually (Supplementary Methods, Supplementary Fig. S3). We found that there were no major differences between these three approaches to calculating survival prediction metrics. Thus, we reported all main results (Fig. 1) using five repetitions of a five-fold CV (Supplementary Methods).

## 4 Discussion

In this work, we introduced *sparsesurv*, a Python package for fitting sparse survival models via KD. *Sparsesurv* provides implementations of the semiparametric AFT and EH models based on kernel-smoothing the profile likelihood. In addition, *sparsesurv* offers easy-to-use KD pipelines that offer flexibility and improved performance compared to non-distilled models, especially for poorly chosen regularization hyperparameters. In particular, KD achieved similar discriminative performance as Elastic Net-based models on the TCGA cancer dataset (Fig. 1A–C) while significantly decreasing sensitivity to the regularization hyperparameter (Fig. 1G and H) and reducing run time (Fig. 1F). Despite these advantages, KD was shown to be quite dependent on the teacher model, which generally represents an upper bound on performance (Fig. 1G and H), which in turn may lead to decreased calibration performance (Fig. 1D). It is worth noting that distilled models tended to display decreased sparsity compared to non-distilled models when considering the optimal regularization hyperparameter (Fig. 1E and Supplementary Table S4). However, this issue can be mitigated to a large extent by employing regularization techniques for the selection of the regularization hyperparameter (Fig. 1E and Supplementary Table S4).

It is important for users to be aware of when the usage of KD can be beneficial for fitting sparse survival models. First, a well-performing teacher model must be chosen. For this purpose, users may rely on benchmark studies that have been performed on both high- and low-dimensional survival data (Herrmann *et al.* 2021, Wissel *et al.* 2022, Zhang *et al.* 2022, Burk *et al.* 2024). Second, if the teacher model performs well, sparse student models generally converge to the performance of the teacher model (Fig. 1G and H). Thus, users can choose sparsity levels almost at will, as the regularization hyperparameter only has little impact on the performance, unlike Elastic Net-based approaches, where it might lead to suboptimal performance and/or require retraining (Fig. 1G and H). In addition, we note that knowledge distillation is still a very active area of research, and future work will likely reveal more on scenarios in which KD can be beneficial and in which it might fail (Stanton *et al.* 2021, Beyer *et al.* 2022, Pavone *et al.* 2023).

Our results also indicate that alternative ways of choosing the regularization hyperparameter, beyond choosing the one that minimizes the cross-validation error, show promise, e.g., the pcvl rule (Ternès *et al.* 2016). However, we observed that another commonly used empirical technique for choosing the regularization hyperparameter, 1se (Friedman *et al.* 2010), namely choosing the highest regularization hyperparameter that is within one standard error of the cross-validation error of the regularization hyperparameter achieving minimum cross-validation error, did not perform as well (Supplementary Fig. S4). Specifically, we found that for both distilled and glmnet approaches, the 1se rule resulted in excessively sparse models, leading to low prediction performance; for many cancer types, we achieved almost only fully sparse models across cross-validation splits (Supplementary Fig. S4, Supplementary Table S2 and Table S3).

Overall, *sparsesurv* offers an easy-to-use Python package for fitting sparse and well-performing Cox PH models while making the choice of the regularization hyperparameter significantly easier. Currently, *sparsesurv* is focused solely on sparse survival models. For possible teacher models, we refer readers to several existing packages that can be readily adapted for use with *sparsesurv* (Tietz *et al.* 2017, Pölsterl 2020).

## Supplementary Material

btae521_Supplementary_Data

## Data Availability

The data underlying this article were provided by the TCGA Research Network: https://www.cancer.gov/tcga. The code of *sparsesurv* is freely available under a BSD 3 license on GitHub (https://github.com/BoevaLab/sparsesurv) and the Python package index (PyPi) (https://pypi.org/project/sparsesurv/).
