# Supplementary material for: sparsesurv: a Python package for fitting sparse survival models via knowledge distillation
Source: Bioinformatics. 2024 Aug 23;40(9):btae521. doi: 10.1093/bioinformatics/btae521 (PMC11387617; doi:10.1093/bioinformatics/btae521)
Supplement: btae521_Supplementary_Data [file btae521_supplementary_data.pdf]

Journal Title Here, 2022, pp. 1–6

doi: DOI HERE
Advance Access Publication Date: Day Month Year
Paper

PAPER

sparsesurv: A Python package for fitting sparse survival models via knowledge distillation

David Wissel [1, 2, 3] Nikita Janakarajan,[1, 4] Julius Schulte [1] Daniel Rowson,[1, 3] Xintian Yuan[1] and Valentina Boeva [1, 3, 5, *]

[1]ETH Zurich, Department of Computer Science, Zurich, Switzerland, [2]University of Zurich, Department of Molecular Life Sciences, Zurich, Switzerland, [3]SIB Swiss Institute of Bioinformatics, Switzerland, [4]IBM Research Europe, Zurich, Switzerland and [5]Université de Paris UMR-S1016 Institut Cochin, Inserm U1016, Paris, France

[*]Corresponding author. valentina.boeva@inf.ethz.ch

FOR PUBLISHER ONLY Received on Date Month Year; revised on Date Month Year; accepted on Date Month Year

Abstract

Motivation: Sparse survival models are statistical models that select a subset of predictor variables while modeling the time until an event occurs, which can subsequently help interpretability and transportability. The subset of important features is often obtained with regularized models, such as the Cox Proportional Hazards model with Lasso regularization, which limit the number of non-zero coefficients. However, such models can be sensitive to the choice of regularization hyperparameter.

Results: In this work, we develop a software package and demonstrate how knowledge distillation, a powerful technique in machine learning that aims to transfer knowledge from a complex teacher model to a simpler student model, can be leveraged to learn sparse survival models while mitigating this challenge. For this purpose, we present sparsesurv, a Python package that contains a set of teacher-student model pairs, including the semi-parametric accelerated failure time and the extended hazards models as teachers, which currently do not have Python implementations. It also contains in-house survival function estimators, removing the need for external packages. Sparsesurv is validated against R-based Elastic Net regularized linear Cox proportional hazards models as implemented in the commonly used glmnet package. Our results reveal that knowledge distillation-based approaches achieve competitive discriminative performance relative to glmnet across the regularization path while making the choice of the regularization hyperparameter significantly easier. All of these features, combined with an sklearn-like API, make sparsesurv an easy-to-use Python package that enables survival analysis for high-dimensional datasets through fitting sparse survival models via knowledge distillation.

Availability: sparsesurv is freely available under a BSD 3 license on GitHub (https://github.com/BoevaLab/sparsesurv) and The Python Package Index (PyPi) (https://pypi.org/project/sparsesurv/).

Contact: valentina.boeva@inf.ethz.ch

Supplementary information: Supplementary data are available at *Bioinformatics* online.

Introduction

Survival analysis, or modeling the time until an event of interest occurs, has been one of the primary statistical tools utilized by clinicians and researchers to determine potential associations of covariates with patient survival [Kalbfleisch and Prentice, 2011]. One of the most widely used models for survival analysis is the Cox Proportional Hazards (Cox PH) due to its comprehensible mechanism of linking covariate effects to the hazard and survival functions [Cox, 1972, Breslow, 1972]. Publicly available cancer cohorts, such as The Cancer Genome Atlas (TCGA), offer molecular data beyond standard clinical characteristics, such as transcriptomic and epigenomic data, to be analyzed using survival analysis methods [Weinstein et al., 2013]. However, due to the large dimensionality of these molecular data, cancer datasets usually have more covariates than patient samples; thus, their analysis requires proper regularization to prevent overfitting. To circumvent this issue and investigate the connections between patient survival and covariates, regularized approaches, such as the Lasso, have been developed for survival analysis, with the additional aim of achieving improved interpretability and ease of use [Tibshirani, 1996, 1997].

Linear regularized methods have contributed to significant advances in survival analysis. However, they are not without their shortcomings. One notable issue is their sensitivity to the choice of regularization hyperparameters, potentially leading to suboptimal performance compared to non-sparse regularized methods like Ridge regression [Ching et al., 2018]. Consequently, it is crucial to develop interpretable and user-friendly approaches that can reproduce the benefits of existing Elastic Net implementations and regularized Cox PH models

© The Author 2022. Published by Oxford University Press. All rights reserved. For permissions, please e-mail: journals.permissions@oup.com

like glmnet while addressing some of their limitations. Existing R packages for sparse survival models tend to focus on specific model classes, such as the Cox PH model in glmnet [Friedman et al., 2010, Simon et al., 2011] and grpreg [Breheny and Huang, 2009, Breheny, 2015, Breheny and Huang, 2015], or the Accelerated Failure Time (AFT) model in penAFT [Suder and Molstad, 2022]. This creates a need for a unifying approach allowing users to train multiple models within one package.

Our implementation is directly inspired by the pre-conditioning approach described by Paul et al. [2008], which would likely be termed Knowledge Distillation (KD) today. In their work, Paul et al. [2008] pointed out the good performance of their pre-conditioning approach when applied to Cox PH models, but it was not rigorously benchmarked against sparse Cox PH models that are fit with regularizers like the Lasso. Throughout this work, we refer to our implemented methods as using KD since we believe this term is more familiar to most readers in the bioinformatics and machine learning communities. We emphasize, however, that the original proposal for fitting sparse semi-parametric survival models in this manner goes back to the proposal of pre-conditioning by Paul et al. [2008].

Here, we propose sparsesurv, an easy-to-use Python package that enables survival analysis for high-dimensional datasets while making the choice of the regularization hyperparameter significantly easier. Our package has two main components. Firstly, we implement variants of the AFT and Extended Hazards (EH) models, each based on kernel-smoothing the profile likelihood [Zeng and Lin, 2007, Tseng and Shu, 2011]. Secondly, we present an easy-to-use pipeline that enables fitting sparse survival models via KD. We implement efficient and numerically stable scoring functions in the form of corresponding model likelihoods and survival function estimators for the Cox PH with Breslow and Efron tie corrections, AFT, and EH models. Through experimental validation, we demonstrate the utility of KD as a valuable tool for sparse survival models. We focus on the Cox PH model as an illustrative example, showcasing how KD can, in some cases, enhance discriminative performance across sparsity levels and simplify the process of selecting the optimal regularization hyperparameter.

Methods

In our experiments, we focus exclusively on right-censored survival analysis (i.e., we disregard truncation and other censoring schemes). The terms "right-censored survival analysis" and "survival analysis" will be used interchangeably hereafter.

Knowledge distillation in sparsesurv

The KD framework separates the process of model estimation from feature selection. It first estimates a model that approximates the outcome well and then estimates a sparse approximation of this first model [Hinton et al., 2015, Stanton et al., 2021]. Our implementation of KD thus consists of two steps. First, a teacher model is fitted to the time-to-event training dataset using the assumptions of an appropriate survival model. The teacher then makes predictions on the entire training dataset. In the second step, these teacher-generated predictions serve as targets to fit the student with a linear regression that is regularized with an appropriate penalty to encourage coefficient sparsity. Our package provides a general class, `KDSurvCV`, that enables the effective separation of teacher and student via sklearn pipelines [Pedregosa et al., 2011, Buitinck et al., 2013].

Implementation details

All functions of sparsesurv are implemented in Python. We use just-in-time compilation via numba for all performance-critical aspects, particularly the calculation of gradients, loss functions, and appropriate survival function estimators [Lam et al., 2015]. We leverage the Application Programming Interface (API) of celer as the sparse regression solver in sparsesurv [Massias et al., 2018]. Sparsesurv uses type hints and is tested using pytest. We implemented our package following the scikit-learn API, which enables the usage of all models with pipelines and other scikit-learn-related features, such as calling `fit` on the model object to train and `predict` for inference. Our package is currently installable from PyPi and GitHub. We refer to the Supplementary for further details on the implementation of sparsesurv.

Experiments

To validate our KD version of the Cox PH model, we performed experiments on 10 transcriptomic datasets from TCGA and compared it to the Elastic Net regularized Cox PH implementation in glmnet. In particular, we used the TCGA datasets from prior work on predicting survival from RNA-seq [Ching et al., 2018]. We focus on the Cox PH model since it is arguably the most commonly used survival model and likewise, glmnet is a commonly used R package to fit survival models. We benchmark both a linear Breslow teacher (Breslow KD) and a neural network-based Breslow teacher (KD Cox-Nnet) for sparsesurv. glmnet, on the other hand, only offers the Breslow approximation and so is only benchmarked on this method for performance comparison. We also included a version of glmnet that tunes the L1 ratio (glmnet tuned (Breslow)) and a version of the sparsesurv Breslow KD model that chooses the regularization hyperparameter to favor sparser models (Breslow KD (pcvl)). We refer to Suppl. Methods and Suppl. Table S1 for further clarity on datasets and Suppl. Methods for experimental details.

Results

The distilled methods implemented in sparsesurv show competitive performance relative to non-distilled models (Fig. 1). In particular, the distilled models (KD Breslow (min) and KD Breslow (pcvl)) slightly outperformed, on average, non-distilled models (glmnet (Breslow)) in terms of Harrell's, Uno's concordance, and Antolini's concordance (Fig. 1A-C), although the improvement was not always statistically significant. When tuning the L1 ratio of the glmnet model, it also performed better than the untuned model, although this came at the cost of decreased sparsity (Fig. 1A-C and Fig. 1E). For the Cox-Nnet teacher, the calibration score showed a small decrease as a result of distillation as measured by the IBS. Otherwise, distilled models and undistilled models performed comparably (Fig. 1D). We observed that distilled Cox PH models were considerably less sparse (Fig. 1E and Suppl. Table S2) compared to the glmnet model, which encountered numerical issues several times and was prone to selecting completely sparse models (Suppl. Table S3). Despite this, KD Breslow (pcvl) was still more sparse than the tuned glmnet model, while achieving comparable prediction performance (Fig. 1A-D). We also found that the

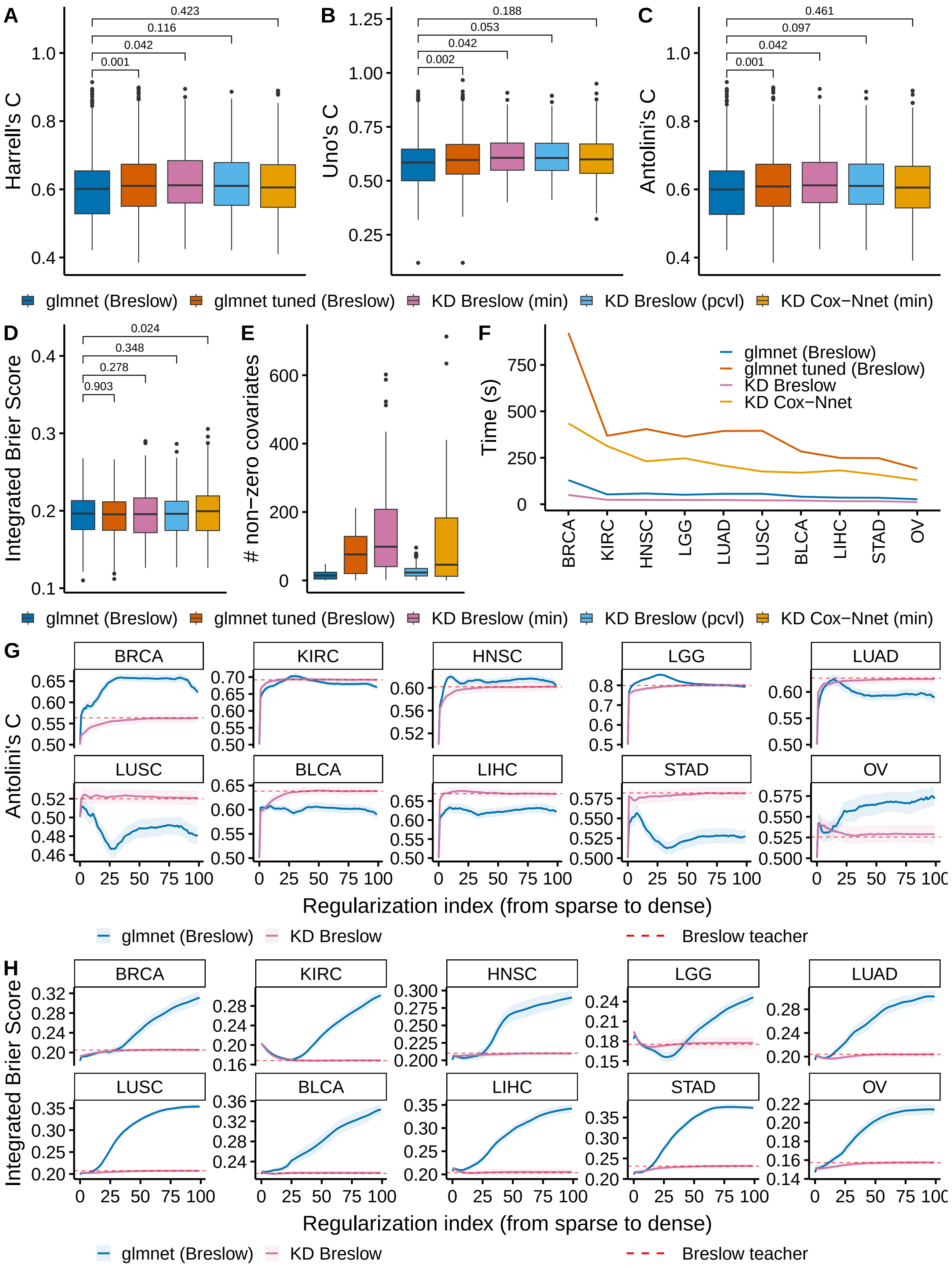
A
B
C
D
E
F
G
H
Harrell's C
Uno's C
Antolini's C
Integrated Brier Score
non−zero covariates
Time (s)
glmnet (Breslow)
glmnet tuned (Breslow)
KD Breslow (min)
KD Breslow (pcvl)
KD Cox−Nnet (min)
KD Breslow
KD Cox−Nnet
Breslow teacher
BRCA
KIRC
HNSC
LGG
LUAD
LUSC
BLCA
LIHC
STAD
OV
Regularization index (from sparse to dense)

Fig. 1. The distilled Elastic Net-regularized Cox PH Breslow model (KD Breslow (min)) performed competitively with a non-distilled Breslow model (glmnet Breslow) in a comparison between sparsesurv and glmnet for sparse Cox PH models on 10 TCGA datasets. The teacher model corresponds to a linear maximum likelihood model with the Breslow tie correction that was trained on a PCA representation obtained from the original datasets (Breslow teacher; Suppl. Methods). Each boxplot represents performances across a 5-fold CV on each dataset of 10 datasets, five times repeated, yielding a total of 250 points in each boxplot. **A-C.** The distilled models performed comparably with the non-distilled Cox PH model trained using glmnet in terms of most concordance measures across all 10 datasets. **D.** The KD models exhibited similar model calibration to the non-distilled Cox PH models. **E.** The non-distilled model exhibited greater sparsity when selecting the regularization hyperparameter that minimizes overall cross-validation error. While the tuned glmnet model also slightly outperformed the untuned glmnet model, it required more variables than a comparable KD model (glmnet tuned (Breslow) vs KD Breslow (pcvl)). **F.** The distilled models were considerably faster than non-distilled models since they can rely on highly optimized sparse solvers for linear regression, such as celer. We do not show both the min and pcvl choices to choose regularization hyperparameters, since they have practically no impact on runtime. **G-H.** The distilled model was more stable in terms of performance than the non-distilled model broadly across the regularization path for most cancer types. This simplifies the selection of the regularization hyperparameter, allowing for more flexibility in inducing specific sparsity levels. "Regularization index" denotes the ordering of the lambda on the regularization path, where zero indicates the first that encourages complete sparsity and 99 indicates the last that encourages minimum sparsity, that is, in our case (see Suppl. Methods). All p-values were calculated with one-sided paired Wilcoxon signed-rank tests. For concordance measures, we applied one-sided statistical tests for the superiority of each model compared to glmnet (Breslow), while for the Integrated Brier Score, we applied one-sided statistical tests for the superiority of glmnet (Breslow) compared to each other model. See Suppl. Methods for further details on statistical significance testing. Confidence bands denote one standard error deviation to either side where applicable. Abbreviations: TCGA, The Cancer Genome Atlas; KD, Knowledge distillation. For cancer-type abbreviations, refer to Suppl. Table S1.

distilled models in sparsesurv were considerably faster than glmnet (Fig. 1F and Suppl. Table S4). The high speed of distilled models can be primarily attributed to the efficient implementations in celer for sparse student models [Massias et al., 2018].

The teachers overall performed similarly to their students, with both types of model slightly outperforming the untuned for the L1-ratio glmnet model in terms of the three concordance measures but not the Integrated Brier Score (IBS) (Fig. S1). The Cox-Nnet student behaved similarly to the student of the PCA-based linear Cox model, with its performance converging to the teacher performance as regularization was lessened, although convergence for the IBS was much more variable relative to the student of the linear teacher (Fig. 1 G-H and Fig. S2). Lastly, we investigated the impact of performing a greater number of cross-validation repetitions (25 instead of 5, see Suppl. Methods) and calculating the concordance measures across all test splits of a CV jointly instead of individually (Suppl. Methods, Suppl. Fig. S3). We found that there were no major differences between these three approaches to calculating survival prediction metrics. Thus, we reported all main results (Fig. 1) using five repetitions of a five-fold CV (Suppl. Methods).

Discussion

In this work, we introduced sparsesurv, a Python package for fitting sparse survival models via KD. Sparsesurv provides implementations of the semiparametric AFT and EH models based on kernel-smoothing the profile likelihood. In addition, sparsesurv offers easy-to-use KD pipelines that offer flexibility and improved performance compared to non-distilled models, especially for poorly chosen regularization hyperparameters. In particular, KD achieved similar discriminative performance as Elastic Net-based models on the TCGA cancer dataset (Fig. 1A-C) while significantly decreasing sensitivity to the regularization hyperparameter (Fig. 1G-H) and reducing run time (Fig. 1F). Despite these advantages, KD was shown to be quite dependent on the teacher model, which generally represents an upper bound on performance (Fig. 1G-H), which in turn may lead to decreased calibration performance (Fig. 1D). It is worth noting that distilled models tended to display decreased sparsity compared to non-distilled models when considering the optimal regularization hyperparameter (Fig. 1E and Suppl. Table S4). However, this issue can be mitigated to a large extent by employing regularization techniques for the selection of the regularization hyperparameter (Fig. 1E and Suppl. Table S4).

It is important for users to be aware of when the usage of KD can be beneficial for fitting sparse survival models. First, a well-performing teacher model must be chosen. For this purpose, users may rely on benchmark studies that have been performed on both high- and low-dimensional survival data [Herrmann et al., 2021, Wissel et al., 2022, Zhang et al., 2022, Burk et al., 2024]. Second, if the teacher model performs well, sparse student models generally converge to the performance of the teacher model (Fig. 1 1G-H). Thus, users can choose sparsity levels almost at will, as the regularization hyperparameter only has little impact on the performance, unlike Elastic Net-based approaches, where it might lead to suboptimal performance and/or require retraining (Fig. 1 1G-H). In addition, we note that knowledge distillation is still a very active area of research, and future work will likely reveal more on scenarios in which KD can be beneficial and in which it might fail [Beyer et al., 2022, Pavone et al., 2023, Stanton et al., 2021].

Our results also indicate that alternative ways of choosing the regularization hyperparameter, beyond choosing the one that minimizes the cross-validation error, show promise, e.g., the pcvl rule [Ternès et al., 2016]. However, we observed that another commonly used empirical technique for choosing the regularization hyperparameter, 1se [Friedman et al., 2010], namely choosing the highest regularization hyperparameter that is within one standard error of the cross-validation error of the regularization hyperparameter achieving minimum cross-validation error, did not perform as well (Suppl. Fig. S4). Specifically, we found that for both distilled and glmnet approaches, the 1se rule resulted in excessively sparse models, leading to low prediction performance; for many cancer types, we achieved almost only fully sparse models across cross-validation splits (Suppl. Fig. S4, Suppl. Table S2 and Suppl. Table S3).

Overall, sparsesurv offers an easy-to-use Python package for fitting sparse and well-performing Cox PH models while making the choice of the regularization hyperparameter significantly easier. Currently, sparsesurv is focused solely on sparse survival models. For possible teacher models, we refer readers to several existing packages that can be readily adapted for use with sparsesurv [Pölsterl, 2020, Tietz et al., 2017].

Competing interests

No competing interest is declared.

Author contributions statement

Conceptualization and methodology: D.W., D.R., J.S., and V.B.; Software implementation: D.W., J.S., N.J., D.R., X.Y.; Validation, formal analysis, and writing the original manuscript: D.W., N.J., J.S., D.R., and V.B. All authors discussed the results and contributed to the final manuscript.

Acknowledgments

The results shown here are in whole or part based upon data generated by the TCGA Research Network: https://www.cancer.gov/tcga. An earlier version of this work was presented at the Research2Clinics workshop at NeurIPS in 2021. We thank all anonymous referees for helping significantly improve our work.

References

John D Kalbfleisch and Ross L Prentice. *The statistical analysis of failure time data*. John Wiley & Sons, 2011.

David R Cox. Regression models and life-tables. *Journal of the Royal Statistical Society: Series B (Methodological)*, 34 (2):187–202, 1972.

Norman E Breslow. Contribution to discussion of paper by dr cox. *Journal of the Royal Statistical Society, Series B*, 34: 216–217, 1972.

John N Weinstein, Eric A Collisson, Gordon B Mills, Kenna R Shaw, Brad A Ozenberger, Kyle Ellrott, Ilya Shmulevich, Chris Sander, and Joshua M Stuart. The cancer genome atlas pan-cancer analysis project. *Nature genetics*, 45(10): 1113–1120, 2013.

Robert Tibshirani. Regression shrinkage and selection via the lasso. *Journal of the Royal Statistical Society Series B: Statistical Methodology*, 58(1):267–288, 1996.

Robert Tibshirani. The lasso method for variable selection in the cox model. *Statistics in medicine*, 16(4):385–395, 1997.

Travers Ching, Xun Zhu, and Lana X Garmire. Cox-nnet: an artificial neural network method for prognosis prediction of high-throughput omics data. *PLoS computational biology*, 14(4):e1006076, 2018.

Jerome Friedman, Trevor Hastie, and Rob Tibshirani. Regularization paths for generalized linear models via coordinate descent. *Journal of statistical software*, 33(1): 1, 2010.

Noah Simon, Jerome Friedman, Trevor Hastie, and Rob Tibshirani. Regularization paths for cox's proportional hazards model via coordinate descent. *Journal of statistical software*, 39(5):1, 2011.

Patrick Breheny and Jian Huang. Penalized methods for bi-level variable selection. *Statistics and its interface*, 2:369–380, 2009.

Patrick Breheny. The group exponential lasso for bi-level variable selection. *Biometrics*, 71:731–740, 2015.

Patrick Breheny and Jian Huang. Group descent algorithms for nonconvex penalized linear and logistic regression models with grouped predictors. *Statistics and Computing*, 25:173–187, 2015.

Piotr M Suder and Aaron J Molstad. Scalable algorithms for semiparametric accelerated failure time models in high dimensions. *Statistics in Medicine*, 41(6):933–949, 2022.

Debashis Paul, Eric Bair, Trevor Hastie, and Robert Tibshirani. " preconditioning" for feature selection and regression in high-dimensional problems. *The Annals of Statistics*, pages 1595–1618, 2008.

Donglin Zeng and DY Lin. Efficient estimation for the accelerated failure time model. *Journal of the American Statistical Association*, 102(480):1387–1396, 2007.

Yi-Kuan Tseng and Ken-Ning Shu. Efficient estimation for a semiparametric extended hazards model. *Communications in Statistics—Simulation and Computation®*, 40(2):258–273, 2011.

Geoffrey Hinton, Oriol Vinyals, and Jeff Dean. Distilling the knowledge in a neural network. *arXiv preprint arXiv:1503.02531*, 2015.

Samuel Stanton, Pavel Izmailov, Polina Kirichenko, Alexander A Alemi, and Andrew G Wilson. Does knowledge distillation really work? *Advances in Neural Information Processing Systems*, 34:6906–6919, 2021.

F. Pedregosa, G. Varoquaux, A. Gramfort, V. Michel, B. Thirion, O. Grisel, M. Blondel, P. Prettenhofer, R. Weiss, V. Dubourg, J. Vanderplas, A. Passos, D. Cournapeau, M. Brucher, M. Perrot, and E. Duchesnay. Scikit-learn: Machine learning in Python. *Journal of Machine Learning Research*, 12:2825–2830, 2011.

Lars Buitinck, Gilles Louppe, Mathieu Blondel, Fabian Pedregosa, Andreas Mueller, Olivier Grisel, Vlad Niculae, Peter Prettenhofer, Alexandre Gramfort, Jaques Grobler, Robert Layton, Jake VanderPlas, Arnaud Joly, Brian Holt, and Gaël Varoquaux. API design for machine learning software: experiences from the scikit-learn project. In *ECML PKDD Workshop: Languages for Data Mining and Machine Learning*, pages 108–122, 2013.

Siu Kwan Lam, Antoine Pitrou, and Stanley Seibert. Numba: A llvm-based python jit compiler. In *Proceedings of the Second Workshop on the LLVM Compiler Infrastructure in HPC*, pages 1–6, 2015.

Mathurin Massias, Alexandre Gramfort, and Joseph Salmon. Celer: a fast solver for the lasso with dual extrapolation. In *International Conference on Machine Learning*, pages 3315–3324. PMLR, 2018.

Moritz Herrmann, Philipp Probst, Roman Hornung, Vindi Jurinovic, and Anne-Laure Boulesteix. Large-scale benchmark study of survival prediction methods using multi-omics data. *Briefings in bioinformatics*, 22(3):bbaa167, 2021.

David Wissel, Nikita Janakarajan, Aayush Grover, Enrico Toniato, María Rodríguez Martínez, and Valentina Boeva. Survboard: standardised benchmarking for multi-omics cancer survival models. *bioRxiv*, pages 2022–11, 2022.

Yunwei Zhang, Germaine Wong, Graham Mann, Samuel Muller, and Jean YH Yang. Survbenchmark: comprehensive benchmarking study of survival analysis methods using both omics data and clinical data. *GigaScience*, 11:giac071, 2022.

Lukas Burk, John Zobolas, Bernd Bischl, Andreas Bender, Marvin N Wright, and Raphael Sonabend. A large-scale neutral comparison study of survival models on low-dimensional data. *arXiv preprint arXiv:2406.04098*, 2024.

Lucas Beyer, Xiaohua Zhai, Amélie Royer, Larisa Markeeva, Rohan Anil, and Alexander Kolesnikov. Knowledge distillation: A good teacher is patient and consistent. In *Proceedings of the IEEE/CVF conference on computer*

vision and pattern recognition, pages 10925–10934, 2022.

Federico Pavone, Juho Piironen, Paul-Christian Bürkner, and Aki Vehtari. Using reference models in variable selection. *Computational Statistics*, 38(1):349–371, 2023.

Nils Ternès, Federico Rotolo, and Stefan Michiels. Empirical extensions of the lasso penalty to reduce the false discovery rate in high-dimensional cox regression models. *Statistics in medicine*, 35(15):2561–2573, 2016.

Sebastian Pölsterl. scikit-survival: A library for time-to-event analysis built on top of scikit-learn. *Journal of Machine Learning Research*, 21(212):1–6, 2020.

Marian Tietz, Thomas J. Fan, Daniel Nouri, Benjamin Bossan, and skorch Developers. *skorch: A scikit-learn compatible neural network library that wraps PyTorch*, July 2017. URL `https://skorch.readthedocs.io/en/stable/`.
